# Effects of insecticides, fipronil and imidacloprid, on the growth, survival, and behavior of brown shrimp *Farfantepenaeus aztecus*

**DOI:** 10.1371/journal.pone.0223641

**Published:** 2019-10-10

**Authors:** Ali Abdulameer Al-Badran, Masami Fujiwara, Miguel A. Mora

**Affiliations:** Department of Wildlife and Fisheries Sciences, Texas A&M University, College Station, Texas, United States of America; Nitte University, INDIA

## Abstract

Increased use of pesticide is causing detrimental effects on non-target species worldwide. In this study, we examined the lethal and sub-lethal effects of fipronil and imidacloprid, two commonly used insecticides, on juvenile brown shrimp (*Farfantepenaeus aztecus*), one of the most commercially and ecologically important species in the United States. The effects of six concentrations of fipronil (0.0, 0.005, 0.01, 0.1, 1.0, and 3.0 μg/L) and six concentrations of imidacloprid (0.0, 0.5, 1.0, 15.0, 34.5, 320.0 μg/L) were tested in a laboratory. We examined five different endpoints: growth, moulting interval, survivorship, behavioral change, and body color change. Growth of shrimp was reduced significantly under higher concentrations of both insecticides. Under fipronil exposure, shrimp in control showed the shortest inter-moult interval (7.57 ± 2.17 day) compared with other treatments; similarly, in the imidacloprid experiment, moulting increased from 8.43 ± 2.52 day in control to 11.95 ± 4.9 day in 0.5 μg/L treatment. Higher concentrations of fipronil (1.0 and 3.0 μg/L) showed a 0.0% survival rate compared with 100% survival in the control and 0.005 μg/L treatment. Under imidacloprid, survivorship decreased from 100% in the control to 33.33% in the 320.0 μg/L treatment. The 96-h LC_50_ of fipronil was 0.12 μg/L, which makes brown shrimp one of the most sensitive invertebrates to the pesticide. Changes in behavior and body color were observed under both insecticides after different durations of exposures depending on concentrations. We conclude that, at the corresponding EPA benchmark concentrations, fipronil had more lethal effects than imidacloprid, and imidacloprid had more sub-lethal effects than fipronil. Both effects are of serious concern, and we suggest monitoring is necessary in estuaries.

## Introduction

The use of chemical pesticides has become critically important to assure both quality and productivity of agricultural products [1, 2] and to control household pests such as termites, fire ants, and mosquitoes [3–5]. However, pesticides also have negative effects on non-target organisms [6–9], which may be considered beneficial organisms. Because chemical pesticides eventually flow into the surface water, it is expected these toxicants to affect aquatic environments more than terrestrial environments [10].

Although these negative effects could be acute (lethal) or chronic (sub-lethal) and vary depending on species [11, 12], the majority of ecotoxicological studies neglect their sub-lethal effects [13, 14] and focus on selected model species, such as daphnia *Daphnia magna* and zebrafish *Danio rerio* [2, 15]. Here, we present the results of experimental studies on the effects of fipronil (5-amino-1-[2, 6-dichloro4-4(trifluoromethyl) phenyl]-4[(trifluoromethyl) sulfinyl]-1H- pyrazole-3-carbonitrile) and imidacloprid (1-[(6-Chloro-3-pyridinyl) methyl]-N-nitro-2-imidazolidinimine), two of the most commonly used chemical pesticides worldwide on brown shrimp *F*. *aztecus*.

Brown shrimp is considered one of the most important commercial species for fisheries along the Atlantic Coast of the southeastern United States and in the Gulf of Mexico [16, 17]. Its commercial landing value was estimated at $166,542 million in 2016 [18]. In addition to its economic importance, brown shrimp also has an essential ecological role as prey for many important fish species in the region [17, 19, 20]. In particular, these shrimp are abundant along the coasts of Texas and Louisiana, U.S.A. and inhabit the estuaries during their juvenile stage [16, 17]. However, because of the increased use of fipronil and imidacloprid in coastal communities, as well as the detection of pesticides residues in estuaries [21], the effects of these insecticides on penaeid shrimp specifically brown shrimp are particularly a serious concern.

Phenylpyrazoles (including fipronil) and neonicotinoids (including imidacloprid) are applied in a large scale, such as protecting plants from agricultural pests, controlling household pests, and controlling parasites on domesticated animals [22]. Presently, they account for approximately one third of the world insecticide market [23]. Although both fipronil and imidacloprid operate by disrupting neural transmission in the central nervous system of invertebrates [23], each product has a different mode of action. Fipronil interferes with the passage of chloride ions by binding to a specific site within the gamma-aminobutyric acid (GABA) receptor, while imidacloprid binds to postsynaptic nicotinic acetylcholine receptors (nAChR) [24, 25]. Compared to other types of insecticides, fipronil and imidacloprid are considered safer because of their low toxicity on fish and mammals. However, fipronil and imidacloprid are very effective on arthropods in small concentrations [26]. Their increased use in recent years [22, 23, 26], moderate to high solubility [27, 28], and persistence in water [29–31] pose a serious concern regarding the potential adverse impacts on non-target aquatic invertebrates.

Fipronil in the environment has been reported in the U.S. and other parts of the world, and its environmental concentrations can be as high as 10.004 μg/L [12, 32–35]. Among the sites where imidacloprid are actively used, its concentration has ranged between 0.016 μg/L and 320.0 μg/L [12, 30, 36–38]. Many of these studies reported the detection of fipronil and imidacloprid, or one or more of their degradation products, in aquatic environments exceeding their chronic levels of the U.S. EPA aquatic life benchmark for invertebrates (0.01 μg/L for both insecticides) [39].

In recent years, many studies have investigated the potential adverse effects of these insecticides on non-target organisms; however, the majority of these studies have focused on a limited number of commercially beneficial terrestrial invertebrates or on selected model organisms in aquatic ecosystems. For example, Pisa, *et al*. [22] found that, of 376 papers reviewed, the majority focused on the effects of fipronil and neonicotinoids on honeybees, and very few studied aquatic invertebrates, particularly marine species.

Both of fipronil and imidacloprid have been detected in aquatic environments in Texas, U.S. in recent years due to their increased use in coastal communities [40]. No previous study published in the peer-reviewed literature has reported the potential effects of these widely-used insecticides on the estuarine-dependent penaeid shrimp such as the brown shrimp *F*. *aztecus*, which are commercially and ecologically important. Therefore, determining the adverse effects of these insecticides on this particular species was critically important. The objective of this study was to evaluate both lethal and sub-lethal effects of the phenylpyrazole fipronil and neonicotinoid imidacloprid on the juvenile brown shrimp under the concentrations observed in the aquatic environment using multiple endpoints: growth (weight and length), moulting, survivorship, and behavioral change. This research complemented many recent studies that highlighted the risks of commonly used pesticides on different species of aquatic organisms, especially not-targeted marine species that have high ecological or commercial impact and limited ecotoxicology data. In addition, this study is the first to investigate the effects of imidacloprid on brown shrimp, and the previous experiments investigating the effects of fipronil on brown shrimp [41] was done only at higher concentrations, where all shrimp individuals died during the first weeks of the experiment and no information about the effects of lower concentrations of fipronil are available.

## Materials and methods

### Collection of brown shrimp

Juvenile brown shrimp were collected on June 12, 2017 (Scientific research permit, SPR-0396-777, Texas Parks and Wildlife Department) from Gangs Bayou in Galveston Bay (on Sportsman Road, N 29.25549; W 94.91575), Texas, using hand nets and a 3-m bag seine of 0.6 cm mesh size. Shrimp were transported to a laboratory in Texas A&M University, College Station, Texas in 45-liter coolers supplied with portable air pumps. After 4–5 hours of water temperature equilibration (between transportation coolers and room temperature), active shrimp were selected for the fipronil experiment (weight 0.57 ± 0.008 g and total length 4.41 ± 0.03 cm) and imidacloprid experiment (weight 0.81 ± 0.01 g and total length 5.31 ± 0.03 cm). After equilibrating temperature, the shrimp were moved to larger plastic tanks of 53-liter filled with artificial brackish water made using Instant Ocean^®^ Sea Salt and dechlorinated tap water. In this study, no vertebrate species, endangered or protected species was involved; no ethical approval was required under Texas A&M University Animal Use Protocol.

### Acclimation period

Shrimp were acclimated for 9 days to laboratory conditions. During the acclimation period, API^®^ Bottom Feeder Shrimp Pellets were used to feed shrimp twice a day to maintain the nutritional requirements of shrimp [42] and to ensure the palatability and acceptability of the pellets by the shrimp. In order to remove excrement and remaining food, the acclimation tanks were cleaned and approximately 50% of their water was replaced on the daily basis. Water quality parameters of acclimation tanks were as follows: dissolved oxygen, 5.6 ± 0.42 mg/L; salinity, 15.50 ± 0.06‰; temperature, 24.53 ± 0.04 °C; and pH, 8.03 ± 0.06 for the fipronil experiment, and dissolved oxygen, 5.5 ± 0.66 mg/L; salinity, 15.73 ± 0.04‰; temperature, 24.43 ± 0.04 °C; and pH, 8.19 ± 0.01 for the imidacloprid experiment. We maintained controlled photoperiod of 12-h: 12-h light: dark cycle. After the acclimation period, shrimp were moved to aquariums used for experiment (S1A Fig).

### Fipronil and imidacloprid experimental solutions

Both fipronil, purity limit ≥ 97% (HPLC), and imidacloprid, purity limit 99.5% (HPLC), were purchased from Fisher Scientific Co. L.L.C., PA, U.S. Based on previously reported concentrations in the environment in other studies (Table 1), six concentrations of fipronil (0.0, 0.005, 0.01, 0.1, 1.0, and 3.0 μg/L) and six concentrations of imidacloprid (0.0, 0.5, 1.0, 15.0, 34.5, 320.0 μg/L) were selected. Three replicates were used for each concentration (treatment).

**Table 1:** Fipronil and imidacloprid concentrations observed in the aquatic environment and reported in previous studies.

Fipronil			Imidacloprid		
Detected concentrations (μg/L)	Location	Reference	Detected concentrations (μg/L)	Location	Reference
0.3–0.8	USA	[21]	0.016	Canada	[37]
1.0	Japan	[12]	0–0.22	Vietnam	[44]
0.28–2.11	USA	[34]	0.2–0.42	Australia	[38]
0.01–4.2	USA	[45]	0–3.3	USA	[46]
3.00–4.54	USA	[47]	1.0–14.0	Slovenia	[36]
0.15–5.0	USA	[48]	17.0–36.0	USA	[49]
0.007–6.0	USA	[50]	49.0	Japan	[12]
0.004–6.41	USA	[32]	320.0	Netherlands	[30]
0.0018–10.004	USA	[33]			
0.09–10.004	USA	[35]			

For each experiment, test solutions of the six nominal concentrations were prepared by a series of dilutions, beginning with mixing a specific amount of the insecticide powder (0.1 g fipronil, and 0.01 g imidacloprid) in 1 liter of artificial brackish water to create a highly homogenized 100 mg/L fipronil suspension and 10 mg/L imidacloprid solution using a magnetic stirrer. For both experiments, the specific dilutions of the nominal concentrations are provided in S1 and S2 Tables. We prepared 21 liters of test solutions for each concentration used in both experiments to fill three aquariums (replicates) of seven liters each. Although the hydrolysis half-life of these compounds at 25°C is much greater than 48 hours, >100 days for fipronil [24] and >30 days for imidacloprid [43], 100% of test solutions were changed every other day to maintain the concentrations relatively constant during the experiment.

### Aquariums and experimental system

Experimental systems were designed in the same way for both fipronil and imidacloprid experiments using new aquariums and other supplies in both experiments. Each system contains 18 glass aquariums of 9.5 liter (30.7 X 15.4 X 20.5 cm), and each aquarium was considered as one replicate (S1A Fig). In order to keep track of shrimp moults individually and to prevent deaths from cannibalism among shrimp, each aquarium was divided into six cells of the same size, and one individual was assigned to each cell (S1B Fig). A screen was placed on each divider to allow water to flow and dissolved oxygen to be distributed evenly among the cells (S1C Fig). The dividers were made of fiberglass screen and polypropylene plates, materials that are commonly used in aquaculture studies.

Each aquarium was filled with 7 liters of test solution, and an air pump was used to provide air. In addition, all aquariums were covered on the top with glass lids to prevent shrimp from escaping. All sides of the aquariums were also covered with aluminum foils to reduce the degradation of the insecticides from exposure to light (S1A Fig). The aquariums were organized in three parallel rows. In each experiment, one of the six treatments was placed randomly in one of the two-column blocks of aquariums.

The fipronil experiment lasted 34 days from June 20, 2017 to July 24, 2017, and the imidacloprid experiment lasted 36 days from June 20, 2017 to July 26, 2017. During both experiments, shrimp were fed on API^®^ Bottom Feeder Shrimp Pellets twice daily. The amounts of food were adjusted weekly (according to the body weight of shrimp) and daily (according to the weight of dead shrimp) using shrimp feeding tables [42]. The temperature (°C), dissolved oxygen (mg/L), salinity (‰), and pH (water quality parameters) were measured with the YSI^®^ Professional plus Multi-parameter Meter every other day.

### Measurements

We conducted all experimental measurements according to the U.S. EPA guidelines [51] and using static-renewal method, with the test solutions being replaced periodically during the experiments. The sample size was determined based on previous studies [52, 53].

#### 1. Weight gain and total length

We measured the weight of shrimp in each treatment every week by gently weighing each shrimp individually. First, shrimp was moved from its cell in the aquarium and placed on paper towel to remove remaining water on the body, and then the weight of shrimp was measured by placing the shrimp in a beaker containing a known amount of water. By weighing shrimp, we were able to monitor the effect of insecticide on growth of shrimp and to gain information for adjusting the amount of food.

Weight gain of shrimp was calculated using the following equation:

$$\%Weight\;gain\;of\;shrimp=\left\{\;\frac{Final\;weight-Initial\;weight}{Initial\;weight}\;\right\}\;x\;100$$ (1)

We also used a caliper (± 0.1 mm) to measure the total length of each shrimp by straighten the body of shrimp carefully on the table and measure the total length from the tip of the head to the end of the tail as shown in S1D Fig.

#### 2. Moulting

Dates of moults of each individual shrimp in both experiments were recorded and used to calculate inter-moult intervals of shrimp in each treatment. Because each shrimp was placed in its own cell, we were able to count the number of days between the two consecutive moults of a particular individual.

#### 3. Shrimp survival and LC_50_

Shrimp movements were monitored multiple times every day, and the numbers of live shrimp were recorded to measure shrimp survivorship during both experiments. Shrimp were considered dead if they did not react or show any response during feeding time, if they did not swim, jump, escape from a net, or move their swimming legs when trying to pick them up for weighing, or if they were in abnormal position such as laying down on the bottom of the aquarium on their back or side without any motion. All dead shrimp were removed, counted, and weighed. Data gained by measuring the survivorship of shrimp were used to estimate the survival rate under both insecticides and the 96-h acute toxicity levels (LC_50_) of fipronil.

#### 4. Behavioral and other physical changes

In both experiments, shrimp were monitored multiple times every day in order to record any abnormal activity. The physical appearance of shrimp in each aquarium was monitored, and changes such as malformations and changes in body color were recorded in comparison with shrimp in the control treatments.

#### 5. Statistical analysis

Non- parametric statistics were used for some measurements when data were not normally distributed. We used Kaplan–Meier estimator to measure the survivorship function of shrimp and non-parametric Log-Rank test to compare the survivorship distribution among treatments. We also used the non-parametric Kruskal-Wallis test followed by the pairwise Wilcoxon rank sum test to compare moulting intervals. LC_50_ value of fipronil was estimated by fitting a generalized linear model to the proportion of individuals died against the pesticide concentration with a logit link and binomial distribution. For all other measurements, we used linear regression analysis and One-way Analysis of Variance (ANOVA) to determine the significance of differences among treatments compared to the control. JMP^®^ Pro 2016 [54] was used to calculate the LC_50_ toxicity test and its 95% confidence intervals, Kruskal-Wallis, ANOVA, and Kaplan–Meier tests, and Microsoft Excel 2016 was used for linear regression analysis and to draw all figures. All of these statistical analyses were conducted at α = 0.05 significance level.

## Results

### Weight gain and total length

**Fig 1:**
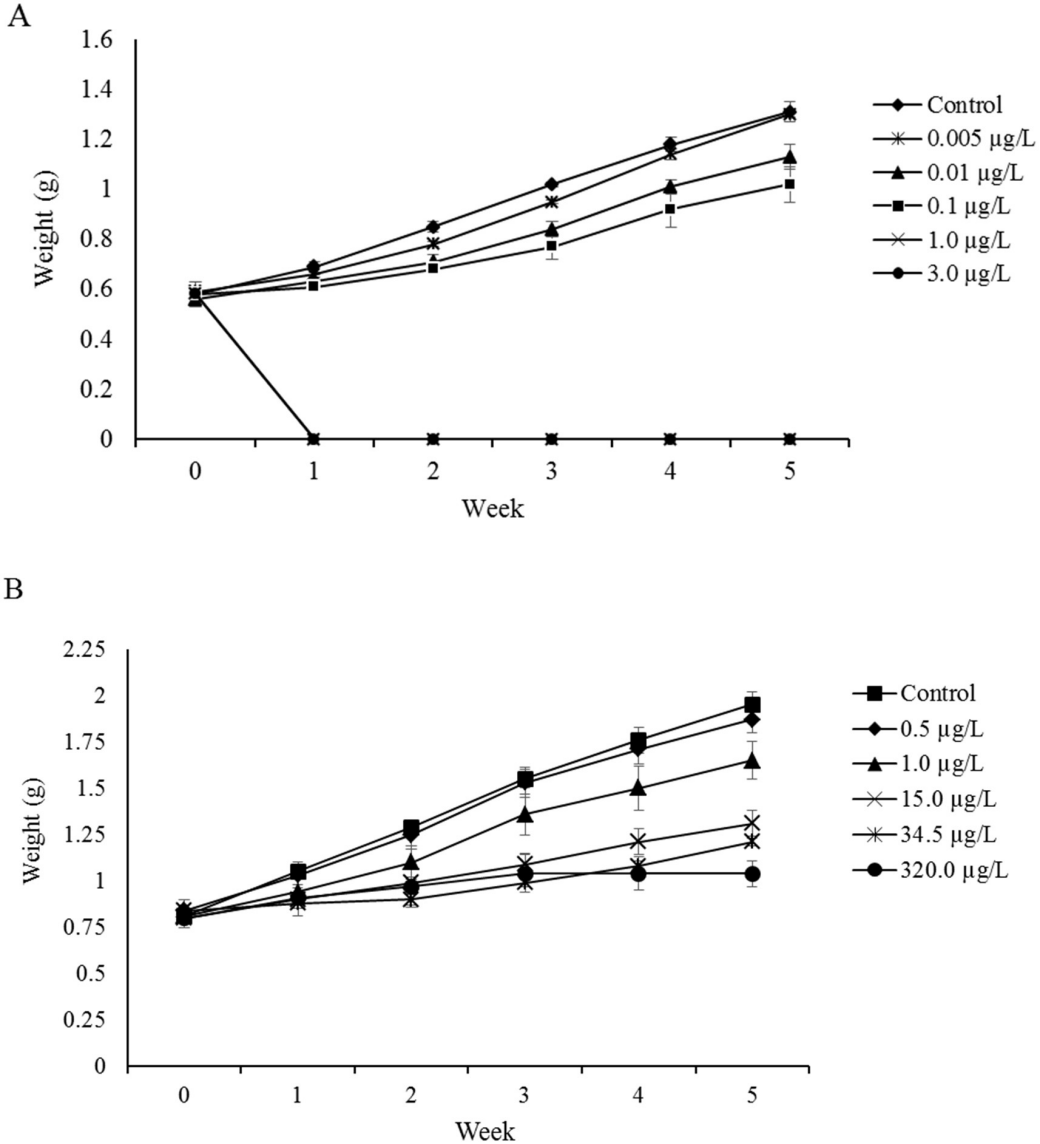
Wet weight of individual juvenile shrimp (g) during 5 weeks of the experiments. The vertical axis is the wet weight (g) of individual shrimp in each treatment, and the horizontal axis is the week from the beginning of experiment. Error bars indicate the standard errors (n = 18 in fipronil exp. and n = 15 in imidacloprid exp.). (**A**) Fipronil experiment, (**B**) Imidacloprid experiment.

The initial weight (at the first day of the experiment) of shrimp exposed to fipronil was not significantly different among all treatments, and the treatment means ranged between 0.56 ± 0.04 g in the 0.01 μg/L treatment and 0.59 ± 0.03 g in the 0.005 μg/L treatment (ANOVA, P = 0.973). Fipronil had a significant effect on the growth of shrimp during the experiment. Final weight of shrimp ranged between 1.02 ± 0.12 g in the 0.1 μg/L treatment and 1.31 ± 0.07 g in the control (0.0 μg/L), which was significantly different from other treatments except the 0.005 μg/L treatment under which the final weight was 1.30 ± 0.03 g (ANOVA, P < 0.0001) (Fig 1A and S3 Table). Fipronil also affected the percent weight gain of shrimp, and significant differences were observed between the control (125.92 ± 28.42%) and the 0.1 μg/L treatment (77.007 ± 21.83% weight gain) (ANOVA, P < 0.0001) whereas there was no significant difference between lower fipronil concentrations (0.005 μg/L and 0.01 μg/L) and the control. The percent weight gain under the latter two concentrations was 120.17 ± 15.16% and 104.18 ± 28.62%, respectively. We could not calculate the final weight and the percent weight gain under the 1.0 μg/L and 3.0 μg/L treatments because all shrimp died during the first days in these treatments (Fig 1A and S3 Table).

Shrimp exposed to imidacloprid also exhibited a reduction in growth during the experiment. The initial mean weight of shrimp ranged between 0.80 ± 0.06 g in the 15.0 μg/L treatment and 0.84 ± 0.06 g in the 34.5 μg/L treatment, and there was no significant difference among all treatments (ANOVA, P = 0.971) (Fig 1B and S4 Table). Final weight ranged between 1.04 ± 0.13 g in the 320.0 μg/L treatment (calculated based on 5 survived shrimp) to 1.95 ± 0.12 g in the control (calculated based on 15 survived shrimp). The final weight under the control was significantly different from all treatments except the 0.5 μg/L treatment (ANOVA, P < 0.0001) (S4 Table). There were also significant differences between the percent weight gain of the control (140.3 ± 16.15%) and three higher imidacloprid concentrations (15.0 μg/L, 34.5 μg/L, and 320.0 μg/L), which showed a reduction in their percent weight gains (64.40 ± 17.14 g%, 44.01 ± 12.09 g%, and 29.48 ± 16.43 g%, respectively)(ANOVA, P = 0.0008), but both 0.5 μg/L and 1.0 μg/L treatments had no significant effect on the percent weight gain compared with the control (S4 Table).

There was no significant difference in the initial body length of shrimp exposed to fipronil, and it ranged between 4.37 ± 0.15 cm in the 1.0 μg/L treatment and 4.46 ± 0.09 cm in the control (0.0 μg/L) treatment (ANOVA, P = 0.794) (S5 Table). Starting from week 1 to week 4, significant differences were observed between the control and all other fipronil concentrations except the 0.005 μg/L treatment (ANOVA, P = 0.004–0.04). At the end of the experiment (fifth length measurement), body length of the control was 6.32 ± 0.10 cm (S5 Table). Body length was not measured in shrimp under both 1.0 μg/L and 3.0 μg/L treatments because the shrimp died during the first days of fipronil exposure.

In the imidacloprid experiment, the initial body length of shrimp was not significantly different among all treatments and ranged between 5.28 ± 0.1 cm in the control and 5.38 ± 0.11 cm in the 34.5 μg/L treatment (ANOVA, P = 0.96) (S6 Table). After the second length measurement (week 2) and until the final measurement (week 5), there were significant differences between the control and other treatments except for the 0.5 μg/L and 1.0 μg/L treatments (ANOVA, P < 0.0001–0.019). At the end of the experiment, body length ranged between 5.91 ± 0.23 cm in the 320.0 μg/L treatment and 7.07 ± 0.12 cm in the control (S6 Table).

### Moulting

**Fig 2:**
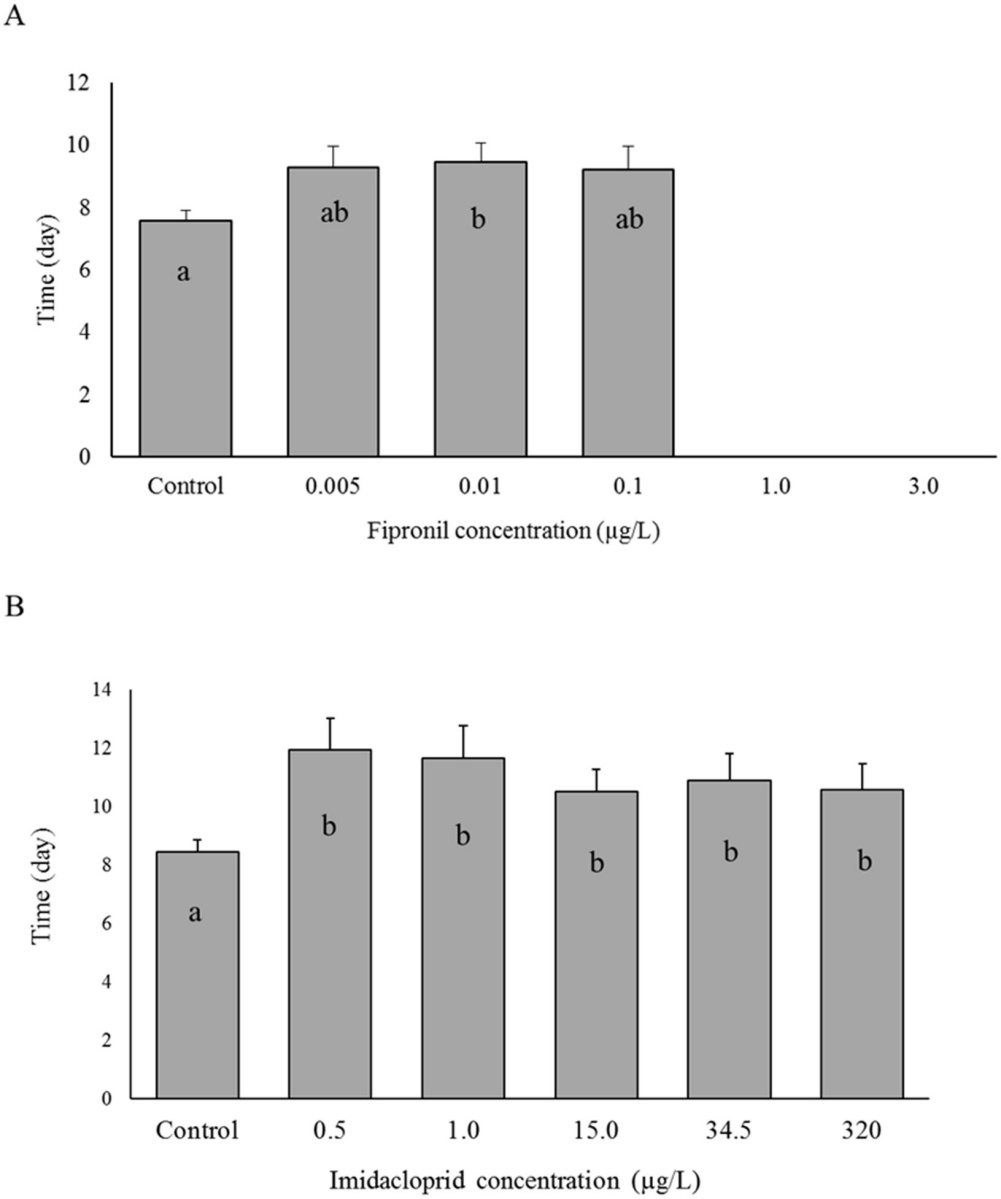
Inter-moult interval of juvenile shrimp under different concentrations of fipronil and imidacloprid. The vertical axis is the time (days) between consecutive moults of the same individual, and the horizontal axis is the six insecticide concentrations (μg/L) including the control. Error bars indicate the standard errors (n = 18 in fipronil exp. and n = 15 in imidacloprid exp.). (**A**) Fipronil experiment, (**B**) Imidacloprid experiment.

In the fipronil experiment, shrimp under the control (0.0 μg/L) showed the shortest inter-moult interval (7.57 ± 2.17 day) compared with other treatments, 0.005 μg/L, 0.01 μg/L, and 0.1 μg/L, which had inter-moult intervals of 9.29 ± 4.22 day, 9.47 ± 2.73 day, and 9.20 ± 2.93 day, respectively (Fig 2A). The inter-moult interval of the control group differed significantly from treatment 0.01 μg/L (P = 0.0117); whereas, there was no difference with treatments 0.005 μg/L and 0.1 μg/L. Shrimp under the 1.0 μg/L and 3.0 μg/L treatments died during the first days of the experiment; thus we could not observe consecutive moults to calculate the inter-moult intervals.

Shrimp under imidacloprid exposure showed a significant difference in inter-moult interval between the control (8.43 ± 2.52 day) and all other treatments (P = 0.0020–0.045). Inter-moult intervals under imidacloprid exposure ranged between 10.50 ± 4.04 day in the 15.0 μg/L treatment and 11.95 ± 4.9 day in the 0.5 μg/L treatment (Fig 2B).

### Shrimp survival and LC_50_

In the fipronil experiment, survivorship of shrimp under higher fipronil concentrations (1.0 μg/L and 3.0 μg/L) decreased rapidly during the first week of the exposure, and all shrimp died by day 6 and day 1, respectively. During the first week, survival rate in the control (100%) was significantly different from that in the 0.1 μg/L treatment (44.44%, P = 0.0006) (Table 2). The survivorship after week 2 differed significantly between the two lowest concentrations (control and 0.005 μg/L) and two higher concentrations (0.01 μg/L and 0.1 μg/L) (P <0.0001) (Table 2). Lethal concentration of fipronil to reach 50% mortality of shrimp within 96 hours (96-h LC_50_) of the juvenile brown shrimp was 0.12 μg/L with 95% confidence intervals 0.06–0.24 (S2 Fig).

**Table 2:** Percentage of individuals alive (mean ± standard deviation) of juvenile shrimp in fipronil experiment starting from day1 to the end of the experiment.

Fipronil concentrations (μg/L)	n	Survival %					
		Day 1	Week 1	Week 2	Week 3	Week 4	Week 5
Control	18	100	100	100	100	100	100
0.005	18	100	100	100	100	100	100
0.01	18	100	77.77 ± 9.62	77.77* ± 9.62	72.21* ± 9.62	72.21* ± 9.62	72.21* ± 9.62
0.1	18	100	44.44* ± 34.69	33.33* ± 16.67	33.33* ± 16.67	33.33* ± 16.67	33.33* ± 16.67
1.0	18	100	0	0	0	0	0
3.0	18	100	0	0	0	0	0

n = number of shrimp individuals in each treatment (6 shrimp per replicate aquarium, 3 aquariums per treatment). Values with star (*) indicate treatment is significantly different from the control (P < 0.0001–0.004).

In the imidacloprid experiment, shrimp showed higher survivorship percentage during the first two weeks compared with those in the fipronil experiment, and there was no significant difference among treatments including the control (Table 3). Starting from week 3, however, significant differences were observed in the survivorship of shrimp in treatments of higher concentrations. At the end of the experiment (36 days), the control treatment showed significant differences in the survival rate (100%) compared with the 15.0 μg/L, 34.5 μg/L, and 320.0 μg/L treatments, which had the survival rates of 66.6%, 40%, and 33.3%, respectively (Kaplan-Meier survival analysis followed by the non-parametric Log-Rank test, P < 0.0001). However, there were no significant differences among the control (100% survival), 0.5 μg/L (93.3% survival) and 1.0 μg/L treatment (86.6% survival) according to the Kaplan-Meier survival analysis (Table 3).

**Table 3:** Percentage of individuals alive (mean ± standard deviation) of juvenile shrimp in imidacloprid experiment starting from day1 to the end of the experiment.

Imidacloprid concentrations (μg/L)	n	Survival %					
		Day 1	Week 1	Week 2	Week 3	Week 4	Week 5
Control	15	100	100	100	100	100	100
0.5	15	100	100	93.33 ± 11.54	93.33 ± 11.54	93.33 ± 11.54	93.33 ± 11.54
1.0	15	100	93.33 ± 11.54	93.33 ± 11.54	93.33 ± 11.54	93.33 ± 11.54	86.66 ± 11.54
15.0	15	100	100	100	100	73.33 ± 23.09	66.66 * ± 30.55
34.5	15	100	100	100	73.33 * ± 23.09	60.0 * ± 20.0	40.0 *
320.0	15	100	100	100	80.0 ± 20.0	60.0 * ± 34.64	33.33 * ± 11.54

n = number of shrimp individuals in each treatment (5 shrimp per replicate aquarium, 3 aquariums per treatment). Values with star (*) indicate treatment is significantly different from the control (P < 0.0001–0.039).

### Behavioral and other physical changes

Swimming and feeding behaviors of brown shrimp under the exposure of fipronil and imidacloprid changed noticeably in comparison with those in the control treatments. These changes were consistent between fipronil and imidacloprid exposures, and a sequence of changes in behaviors was observed from the first day of the exposure (in some treatments) until the death of the affected shrimp. First, affected shrimp became unable to swim normally, and they started exhibiting circle-like movements. After that, shrimp stopped moving and sprawled on the bottom of an aquarium at the same time their swimming legs kept moving involuntary. Then, their swimming legs stopped moving, and they died. Shrimp in the fipronil experiment showed these behavioral changes after only one day of the exposure and even in the lowest concentration (0.005 μg/L). Similarly, shrimp under imidacloprid exposure showed same behavioral changes by day one in all treatments except in the 0.5 μg/L treatment (lowest imidacloprid concentration). Under the lowest imidacloprid concentration, these changes started in day 5. During all of these abnormal swimming behaviors, shrimp exhibited difficulty in feeding, and food remained in aquariums were cleaned routinely.

**Fig 3:**
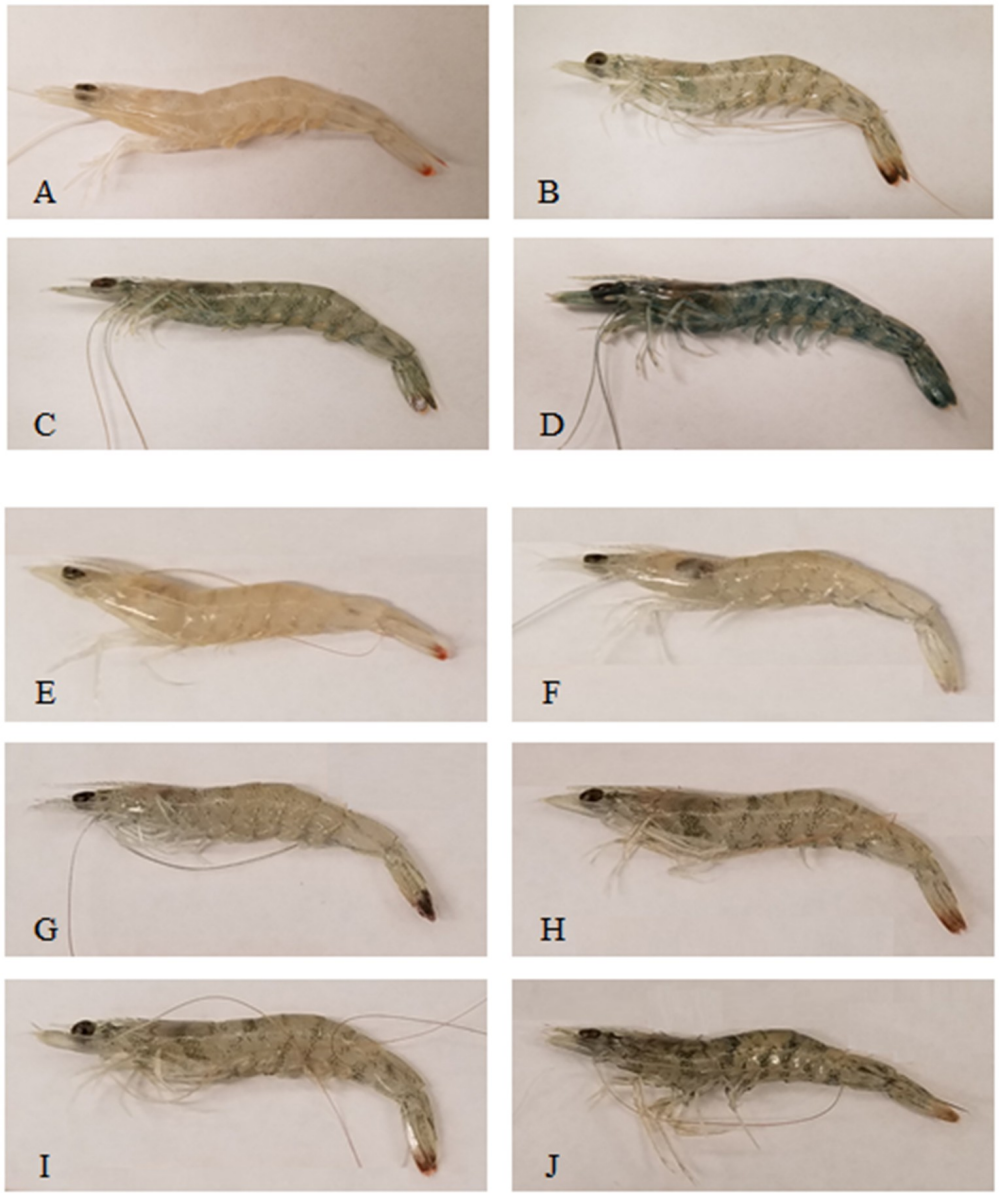
Color change of shrimp in different concentrations (μg/L) of the insecticides at the end of the experiments. (**A**) Control 0.0 μg/L fipronil, (**B**) 0.005 μg/L fipronil, (**C**) 0.01 μg/L fipronil, (**D**) 0.1 μg/L fipronil, (**E**) Control 0.0 μg/L imidacloprid, (**F**) 0.5 μg/L imidacloprid, (**G**) 1.0 μg/L imidacloprid, (**H**) 15.0 μg/L imidacloprid, (**I**) 34.5 μg/L imidacloprid, (**J**) 320.0 μg/L imidacloprid.

At the end of the experiments, visible changes in color were noticed in shrimp bodies under the exposure to either pesticide. Fig 3 shows the changes in color of shrimp from the normal bright color under the control to gray and dark body color of shrimp in treatments of high fipronil and imidacloprid concentrations. In the fipronil experiment, shrimp exposed to higher concentrations (1.0 μg/L and 3.0 μg/L) died during the first days and did not show color changes.

### Water quality

In both fipronil and imidacloprid experiments, all water quality parameters were within suitable ranges that fit the environmental requirements of brown shrimp [55]. Statistical analysis of these parameters showed no significant differences among treatments during the experiments. In the fipronil experiment, the mean values of water quality parameters were the following: temperature, 24.12 ± 0.06 °C; dissolved oxygen (DO), 5.82 ± 0.41 mg/L, salinity, 15.02 ± 0.14‰; and pH, 8.01 ± 0.06 (S7 Table). In the imidacloprid experiment, mean values were the following: temperature, 24.34 ± 0.03 °C; dissolved oxygen (DO), 5.87 ± 0.06 mg/L, salinity, 15.48 ± 0.08‰; and pH, 8.15 ± 0.05 (S8 Table).

## Discussion

In the present study, all nominal concentrations of fipronil and imidacloprid were within the range of the concentrations reported in recent studies [12, 30, 35, 37] for both insecticides (Table 1). Our results showed that, for both insecticides, significant differences were observed in the final weight and final length (length at week 5) of shrimp under many of these concentrations compared with the control (S3–S6 Tables) suggesting the insecticides have effects to reduce growth of shrimp. Reduction in growth of aquatic arthropods resulting from contaminants has been demonstrated in several other studies: e.g. the Glyphosate-based herbicide (Roundup^®^) on freshwater shrimp *Caridina nilotica* [56], petroleum hydrocarbons from oil spill on juvenile brown shrimp *F*. *aztecus* and white shrimp *L*. *setiferus* [57], and imidacloprid on midge *Chironomus tentans*, amphipod *Hyalella azteca* [58], and harlequin fly *Chironomus riparius* [59]. However, we note that growth of juvenile blue crabs *Callinectes sapidus* showed a significant increase under all treatments of fipronil compared to the control in a short-term (96-h) experiment [60], suggesting the effects of insecticides on growth may not be always negative.

Many potential reasons exist for the reduced growth of juvenile brown shrimp under fipronil and imidacloprid exposures in our study. For example, organisms in polluted environments use the metabolic energy to detoxify the contaminant. Therefore, this will affect their growth performance by affecting the metabolism of protein and carbohydrate [61]. Shrimp is known to derive energy more efficiently from protein compared with carbohydrates and lipids [62]; consequently, fipronil and imidacloprid in our study may have affected brown shrimp growth by reducing protein levels in bodies of those under exposure compared with shrimp in the control, which in turn affected their growth at the end of the experiment (S3–S6 Tables). Alternatively, the reduction in growth may also be caused by reduced feeding. Both insecticides are neurotoxins, which act by disrupting the central nervous system activity of exposed arthropods either by blocking the chloride channels at the gamma-aminobutyric acid (GABA) by fipronil [63] or by binding strongly to the nicotinic acetylcholine receptor (nAChR) by imidacloprid [64]. These effects on the nervous system activities may suppress feeding of invertebrates [65]. Regardless of the underlying mechanisms, the reduced growth from the insecticide is a great concern because the survival of juvenile brown shrimp is thought to be size dependent [66] and the abundance of adult white shrimp, which has very similar life history as brown shrimp has been demonstrated to be very sensitive to survival during a juvenile stage [67].

Moulting in arthropods is useful endpoint to test sub-lethal exposure of chemicals, and considered one of the most important physiological processes for these animals because in order to grow normally they have to cast their exoskeleton periodically [68, 69]. Moulting process is regulated by hormones and nervous system secretions; therefore, it is susceptible to the negative effects of endocrine disrupting chemicals EDCs including many pesticides [69], such as fipronil [70] or those who act like EDCs such as imidacloprid [71]. In our study, both fipronil and imidacloprid affected moulting of brown shrimp with significant differences between the control and other treatments. Inter-moult interval of shrimp under fipronil exposure was significantly delayed under the 0.01 μg/L treatment compared with the control (Fig 2A). This result is consistent with our previous study, which showed the prolonged inter-moult intervals under the 0.1 μg/L and 1.0 μg/L treatments compared with the control [41]. In our previous study, we evaluated the adverse effects of higher concentrations of fipronil (0.0, 0.1, 1.0, 3.0, 6.4, 10.0 μg/L) on brown shrimp juveniles, and because of the higher concentrations used, the exposure to all of the fipronil treatments resulted in all individuals dying before the end of the experiment. Under imidacloprid exposure in the current study, shrimp under all treatments showed a significant delay (P = 0.0020–0.045) in their inter-moult intervals (10.89 ± 3.97 day in 34.5 μg/L to 11.95 ± 4.90 day in 0.5 μg/L) compared with shrimp in control (8.43 ± 2.52 day) (Fig 2B). Many studies have reported similar delay in moulting of marine and freshwater arthropods after exposing them to different pesticides [41]. Such delay in moulting of shrimp may be linked to the reduction in growth and suggests that normal development of shrimp was affected even under concentrations below chronic level (0.01 μg/L) of fipronil.

Our study showed that survival of juvenile brown shrimp was concentration-dependent under both insecticides (Tables 2 and 3). Fipronil and imidacloprid caused significant lethal and sub-lethal effects on shrimp especially in higher concentrations. Under fipronil exposure, all shrimp died during first few days in the 1.0 μg/L and 3.0 μg/L treatments and survivorship declined significantly (P < 0.0001) under the 0.1 μg/L (33.33%) and 0.01 μg/L (72.21%) treatments compared with the control (100% survival) (Table 2). Under imidacloprid exposure, survivorship declined significantly (P < 0.0001) in the 320.0 μg/L (33.33%), 34.5 μg/L (40.0%), and 15.0 μg/L (66.66%) treatments compared with control (100%) (Table 3).

The nominal 96-h LC_50_ of fipronil for brown shrimp was 0.12 μg/L (0.06–0.24). This suggests brown shrimp is one of the most sensitive crustaceans to fipronil among all aquatic invertebrates studied so far. This 96-h LC_50_ for brown shrimp is less than that for estuarine mysid shrimp *Neomysis americana* LC_50_ = 0.14 μg/L reported in Gan, *et al*. [33]. Other sensitive marine invertebrates include estuarine grass shrimp *Palaemonetes pugio* with 96-h LC_50_ of 0.32 μg/L [72, 73], estuarine copepod *Amphiascus tenuiremis* with 96-h LC_50_ of 6.8 μg/L [21], and estuarine Chinese mitten crab *Eriocheir sinensis* with 96-h LC_50_ of 8.56 μg/L [52]. Many previous studies reported the greater sensitivity of marine invertebrates to fipronil compared with freshwater invertebrates, such as the copepod *Acanthocyclops robustus* with 48-h LC_50_ of 194.2 μg/L [74], the water flea *D*. *magna* with 48-h LC_50_ of 190.0 μg/L [27], and the red swamp crayfish *Procambarus clarkia* with 96-h LC_50_ of 163.5 μg/L [73]. As for imidacloprid, we could not measure the LC_50_ for brown shrimp because there was not enough mortality in shrimp during the first 96 hours of the exposure under the concentrations used in this study.

We also note that the LC_50_ of fipronil measured in this study (0.12 μg/L) was below the LC_50_ measured in the previous study in 2016, 1.3 μg/L [41]. This may be because of the difference in the temperature between the two studies: 20.84°C ± 0.24 (in 2016 study) and 24.12°C ± 0.06 (in present study). Although temperature was increased, these water temperatures are considered within the optimum temperature range for the brown shrimp development 18°C and 25°C [55]. We increased water temperature from the previous study because we expected shrimp to experience higher temperature during summer months in estuaries and previous shrimp experiments by others were often conducted at higher temperature e.g. [75–77]. Effect of temperature on toxicity of pesticides was observed in other studies, for example, Russo, *et al*. [78] noted that, of many environmental parameters investigated, temperature was the only parameter that magnified the effect of pesticide exposure on the crustacean *Gammarus pulex* in streams. Willming *et al*. [79] also reported that the 10 days LC_50_ for the crustacean *H*. *azteca* exposed to the fungicide chlorothalonil was lower under the fluctuating temperature regime compared with that under the constant temperature regime. We suggest further studies to investigate the effects of temperature on the effects of insecticides in the future study as the temperature in the subtropical estuaries (habitat for juvenile brown shrimp) can change greatly among seasons.

Behavioral changes are useful biomarkers to evaluate sub-lethal exposure effects of contamination [80]. Long-term behavioral changes can be detected even at low doses of pesticides, and the behavior can reveal a great deal about the systems and processes influenced by pesticides [81]. In the current study, shrimp in both experiments exhibited behavioral changes such as restricted swimming and mobility, paralysis, and feeding delay, starting from the first day of exposure even in treatments of low fipronil concentrations (0.005 and 0.01 μg/L) whereas those in treatment of low imidacloprid concentration (0.5 μg/L) did not show any behavioral changes until day 5 of the exposure. Similar behavioral changes were reported in studies that tested the effect of fipronil, imidacloprid, and other pesticides on aquatic invertebrates. For example, Overmyer *et al*. [26] observed abnormal behavior and muscle control in the aquatic insect *Simulium vittatum* under all tested fipronil and imidacloprid concentrations. Similarly, Stratman *et al*. [82] showed that the chironomid midge *Cricotopus betis* exposed to different concentrations of fipronil exhibited abnormal behaviors such as movement restriction and feeding reduction at all tested concentrations. Behavioral changes could be direct consequences of pesticides on the central nervous system of organisms [83]. These changes may have substantial ecological effects to the organisms by shifting them to unfavorable habitats or even by making them more sensitive to predators, and eventually leading to indirect lethal responses of pollutants at sub-lethal levels [69]. In particular, the major mortality of juvenile brown shrimp is considered to be predations [66], and small effects on their behavior may cause substantial reduction in their in situ mortality rate.

In both experiments, shrimp showed darker body color in treatments of higher concentrations of insecticides compared with those in control, which had normal bright color as they are in nature. The color also appears to be a concentration-dependent, and shrimp under fipronil exposure were much darker than those under imidacloprid exposure. In other study, Martinez *et al*. [84] reported that Pacific white shrimp *Litopenaeus vannamei* exposed to low concentrations of heavy metals such as copper were significantly redder than those in controls. In shrimp and other crustaceans, many environmental factors are known to affect body color by affecting pigment dispersion within the chromatophores [85], which are regulated by neurosecreted hormones [86]. However, in our study, factors such as light intensity, background color, and temperature which are known to have an effect on body color of shrimp were carefully controlled. Color changes under fipronil and imidacloprid insecticides were observed in the exoskeleton and abdominal muscle of juvenile brown shrimp as shown in Fig 3. Body color may be used as an indicator of the health of shrimp [84] and environment [87]; consequently, we suggest that color change of penaeid shrimp needs more investigation because it could be used as an indicator of long-term effects of sub-lethal exposure to environmental neurotoxins such as fipronil, imidacloprid or other commonly used pesticides.

## Conclusions

In summary, fipronil and imidacloprid had both lethal and sub-lethal effects on brown shrimp at concentrations that have been reported in the natural environment. According to the 96-h LC_50_ of fipronil, brown shrimp is one of the most sensitive invertebrate species to fipronil exposure among all marine and freshwater species studied to date. Although brown shrimp exhibited less lethal effects on imidacloprid than fipronil under the U.S. EPA benchmark concentrations, sub-lethal effects such as delayed moulting and reduced growth were significant. Both types of effects are of serious concern because of the commercial and ecological importance of brown shrimp, their dependency on estuaries, and increased use of these pesticides in coastal areas. We recommend continuous monitoring of both chemicals, and it may also be necessary to reduce the use of these chemicals when brown shrimp are abundant in estuaries.

## Supporting information

S1 Fig Experimental systems and the aquariums used in fipronil and imidacloprid experiments. (**A**) System of 18 glass aquariums covered with aluminum foil sheets and glass lids; (**B**) aquarium was divided into six cells of same size; (**C**) dividers made of polypropylene plates and fiberglass screen to distribute the dissolved oxygen among the cells; (**D**) total length of shrimp measured every week during the experiments.
(DOCX)

S2 Fig Lethal concentration of fipronil to reach 50% mortality of shrimp within 96 hours (96-h LC_50_) of the juvenile brown shrimp. (DOCX)

S1 Table Dilution procedures for all nominal fipronil concentrations used in the experiment. For steps 1 and 2, magnetic stirrer was used to homogenize the mixture.
(DOCX)

S2 Table Dilution procedures for all nominal imidacloprid concentrations used in the experiment. For steps 1 and 2, magnetic stirrer was used to homogenize the mixture.
(DOCX)

S3 Table Initial weight (g), final weight (g), and percent weight gain (mean ± standard deviation) of juvenile shrimp under different concentrations of fipronil. n = number of shrimp in each treatment at the measurement time. Values were calculated based on the wet weight per individual shrimp. Means in columns not sharing the same letter are significantly different (ANOVA, P < 0.05).
(DOCX)

S4 Table Initial weight (g), final weight (g), and percent weight gain (mean ± standard deviation) of juvenile shrimp under different concentrations of imidacloprid. n = number of shrimp in each treatment at the measurement time. Values were calculated based on the wet weight per individual shrimp. Means in columns not sharing the same letter are significantly different (ANOVA, P < 0.05).
(DOCX)

S5 Table Length (cm) of juvenile shrimp (mean ± standard deviation) exposed to fipronil over five weeks. n = number of shrimp in each treatment. Means in columns not sharing the same letter are significantly different (ANOVA, P < 0.05).
(DOCX)

S6 Table Length (cm) of juvenile shrimp (mean ± standard deviation) exposed to imidacloprid over five weeks. n = number of shrimp in each treatment. Means in columns not sharing the same letter are significantly different (ANOVA, P < 0.05).
(DOCX)

S7 Table Water quality parameters of shrimp aquariums during 34 days of fipronil experiment. Values are Mean ± standard deviation for each parameter of all fipronil concentrations. Treatment 1.0 μg/L has no standard deviation because the number of aquariums was reduced to 1 due to deaths of shrimp during first days of the experiment. Treatment 3.0 μg/L has no water quality parameters because all shrimp died during the first day of the exposure.
(DOCX)

S8 Table Water quality parameters of shrimp aquariums during 36 days of imidacloprid experiment. Values are Mean ± standard deviation for each parameter of all imidacloprid concentrations.
(DOCX)

## References

[1] Oerke EC, Dehne HW. Safeguarding production—losses in major crops and the role of crop protection. Crop Protection. 2004;23(4):275–85.

[2] Hayasaka D, Korenaga T, Suzuki K, Saito F, Sanchez-Bayo F, Goka K. Cumulative ecological impacts of two successive annual treatments of imidacloprid and fipronil on aquatic communities of paddy mesocosms. Ecotoxicol Environ Saf. 2012;80:355–62. 10.1016/j.ecoenv.2012.04.004 . 22521688

[3] Elliott R, Barnes JM. Organophosphorus Insecticides for the Control of Mosquitos in Nigeria. Trials with Fenthion and Malathion Conducted by the WHO Insecticide Testing Unit in 1960–61. Bull Org mond Sante. 1963;28:35–54. PMC2554662 20604136

[4] Aktar MW, Sengupta D, Chowdhury A. Impact of pesticides use in agriculture: their benefits and hazards. Interdiscip Toxicol. 2009 3;2(1):1–12. 10.2478/v10102-009-0001-7 . 21217838 PMC2984095

[5] Drees BM. How to Select, Apply, and Develop Insecticides for Imported Fire Ant Control. Texas A&M AgriLife Extension Service. 2014:8 p.

[6] Clasen B, Loro VL, Cattaneo R, Moraes B, Lopes T, de Avila LA, et al Effects of the commercial formulation containing fipronil on the non-target organism Cyprinus carpio: implications for rice-fish cultivation. Ecotoxicol Environ Saf. 2012 3;77:45–51. . 22078114 10.1016/j.ecoenv.2011.10.001

[7] Roessink I, Merga LB, Zweers HJ, Van den Brink PJ. The neonicotinoid imidacloprid shows high chronic toxicity to mayfly nymphs. Environ Toxicol Chem. 2013;32(5):1096–100. 10.1002/etc.2201 . 23444274

[8] Goulson D. REVIEW: An overview of the environmental risks posed by neonicotinoid insecticides. Journal of Applied Ecology. 2013;50(4):977–87.

[9] Krupke CH, Long EY. Intersections between neonicotinoid seed treatments and honey bees. Curr Opin Insect Sci. 2015;10:8–13. 10.1016/j.cois.2015.04.005 29588017

[10] Pritchard JB. Aquatic toxicology: past, present, and prospects. Environ Health Perspect. 1993;100:249–57. 10.1289/ehp.93100249 8354173 PMC1519578

[11] Laboy-Nieves EN, Schaffner FC, Abdelhadi AH, Goosen MFA. Environmental Management, Sustainable Development and Human Health. London, UK: CRC Press/Balkema; 2009.

[12] Hayasaka D, Korenaga T, Sanchez-Bayo F, Goka K. Differences in ecological impacts of systemic insecticides with different physicochemical properties on biocenosis of experimental paddy fields. Ecotoxicology. 2012 1;21(1):191–201. 10.1007/s10646-011-0778-y . 21877228

[13] Shaw JR, Pfrender ME, Eads BD, Klaper R, Callaghan A, Sibly RM, et al Daphnia as an emerging model for toxicological genomics. Adv Exp Biol. 2008;2:165–328.

[14] Abbott LC. Selecting optimal animal models to investigate environmental toxicology. Poult Fish Wildl Sci. 2013;1.

[15] Dai YJ, Jia YF, Chen N, Bian WP, Li QK, Ma YB, et al Zebrafish as a model system to study toxicology. Environ Toxicol Chem. 2014 1;33(1):11–7. 10.1002/etc.2406 . 24307630

[16] Ditty JG. Young of Litopenaeus setiferus, Farfantepenaeus aztecus and F. duorarum (Decapoda: Penaeidae): a re-assessment of characters for species discrimination and their variability. J Crustac Biol. 2011;31(3):458–67.

[17] Montero JT, Chesney TA, Bauer JR, Froeschke JT, Graham J. Brown shrimp (Farfantepenaeus aztecus) density distribution in the Northern Gulf of Mexico: an approach using boosted regression trees. Fish Oceanogr. 2016;25(3):337–48.

[18] NMFS. Commercial fisheries statistics. National Marine Fisheries Service. https://www.st.nmfs.noaa.gov/commercial-fisheries/commercial-landings/annual-landings-with-group-subtotals/index (Accessed: 5th July 2018). 2017.

[19] Sheridan PF, Ray SM. Report of the workshop on the ecological interactions between shrimp and bottomfishes, April 1980. National Marine Fisheries Service (NMFS). Southeast Fisheries Center. Galveston, TX; 1981.

[20] Fujiwara M, Zhou C, Acres C, Martinez-Andrade F. Interaction between Penaeid Shrimp and Fish Populations in the Gulf of Mexico: Importance of Shrimp as Forage Species. PloS one. 2016;11(11):e0166479 10.1371/journal.pone.0166479 . 27832213 PMC5104333

[21] Chandler GT, CARY TL, Volz DC, Walse SS, Ferry JL, KLOSTERHAUS SL. Fipronil Effects on Estuarine Copepod (Amphiascus Tenuiremis) Development, Fertility, and Reproduction: A Rapid Life-Cycle Assay in 96-Well Microplate Format. Environmental Toxicology and Chemistry. 2004;23(1):117–24. 10.1897/03-124 14768875

[22] Pisa LW, Amaral-Rogers V, Belzunces LP, Bonmatin JM, Downs CA, Goulson D, et al Effects of neonicotinoids and fipronil on non-target invertebrates. Environ Sci Pollut Res Int. 2015;22(1):68–102. 10.1007/s11356-014-3471-x 25223353 PMC4284392

[23] Simon-Delso N, Amaral-Rogers V, Belzunces LP, Bonmatin JM, Chagnon M, Downs C, et al Systemic insecticides (neonicotinoids and fipronil): trends, uses, mode of action and metabolites. Environ Sci Pollut Res (). 2015;22:5–34. 10.1007/s11356-014-3470-y PMC4284386 25233913

[24] Gunasekara AS, Truong T, Goh KS, Spurlock F, Tjeerdema RS. Environmental fate and toxicology of fipronil. Journal of Pesticide Science. 2007;32(3):189–99.

[25] Mortensen SR, Holmsen JD, Weltje L. Fipronil should not be categorized as a "systemic insecticide": a reply to Gibbons et al. (2015). Environmental science and pollution research international. 2015 11;22(21):17253–4. 10.1007/s11356-015-4719-9 . 26002371

[26] Overmyer JP, Mason BN, Armbrust KL. Acute toxicity of imidacloprid and fipronil to a nontarget aquatic insect, Simulium vittatum Zetterstedt cytospecies IS-7. Bull Environ Contam Toxicol. 2005;74(5):872–9. 10.1007/s00128-005-0662-7 16097320

[27] USEPA. New pesticide fact sheet: fipronil. United States Environmental Protection Agency. Office of Prevention, Pesticides and Toxic Substances. Washington DC. USA; 1996.

[28] Raby M, Zhao X, Hao C, Poirier DG, Sibley PK. Chronic effects of an environmentally-relevant, short-term neonicotinoid insecticide pulse on four aquatic invertebrates. The Science of the total environment. 2018 10 15;639:1543–52. 10.1016/j.scitotenv.2018.05.259 . 29929317

[29] Tisler T, Jemec A, Mozetic B, Trebse P. Hazard identification of imidacloprid to aquatic environment. Chemosphere. 2009;76(7):907–14. 10.1016/j.chemosphere.2009.05.002 . 19505710

[30] Van Dijk TC, Van Staalduinen MA, Van der Sluijs JP. Macro-invertebrate decline in surface water polluted with imidacloprid. PloS one. 2013;8(5):e62374 10.1371/journal.pone.0062374 . 23650513 PMC3641074

[31] McMahen RL, Strynar MJ, McMillan L, DeRose E, Lindstrom AB. Comparison of fipronil sources in North Carolina surface water and identification of a novel fipronil transformation product in recycled wastewater. The Science of the total environment. 2016 11 1;569–570:880–7. 10.1016/j.scitotenv.2016.05.085 . 27378152

[32] Mize SV, Porter SD, Demcheck DK. Influence of fipronil compounds and rice-cultivation land-use intensity on macroinvertebrate communities in streams of southwestern Louisiana, USA. Environmental pollution. 2008 3;152(2):491–503. 10.1016/j.envpol.2007.03.021 . 17706328

[33] Gan J, Bondarenko S, Oki L, Haver D, Li JX. Occurrence of fipronil and its biologically active derivatives in urban residential runoff. Environ Sci Technol. 2012;46(3):1489–95. 10.1021/es202904x . 22242791

[34] Ensminger MP, Budd R, Kelley KC, Goh KS. Pesticide occurrence and aquatic benchmark exceedances in urban surface waters and sediments in three urban areas of California, USA, 2008–2011. Environmental monitoring and assessment. 2013 5;185(5):3697–710. 10.1007/s10661-012-2821-8 . 22899460

[35] Ruby A. Review of pyrethroid, fipronil and toxicity monitoring data from california urban watersheds. California Stormwater Quality Association (CASQA). 2013:90 p.

[36] Jemec A, Tisler T, Drobne D, Sepcic K, Fournier D, Trebse P. Comparative toxicity of imidacloprid, of its commercial liquid formulation and of diazinon to a non-target arthropod, the microcrustacean Daphnia magna. Chemosphere. 2007;68(8):1408–18. 10.1016/j.chemosphere.2007.04.015 . 17524455

[37] Main AR, Headley JV, Peru KM, Michel NL, Cessna AJ, Morrissey CA. Widespread use and frequent detection of neonicotinoid insecticides in wetlands of Canada’s Prairie Pothole Region. PloS one. 2014;9(3):e92821 10.1371/journal.pone.0092821 . 24671127 PMC3966823

[38] Hook SE, Doan H, Gonzago D, Musson D, Du J, Kookana R, et al The impacts of modern-use pesticides on shrimp aquaculture: An assessment for north eastern Australia. Ecotoxicol Environ Saf. 2018 2;148:770–80. 10.1016/j.ecoenv.2017.11.028 . 29190596

[39] USEPA. Aquatic life benchmarks for pesticide registration Washington, DC. https://www.epa.gov/pesticide-science-and-assessing-pesticide-risks/aquatic-life-benchmarks-and-ecological-risk: Environmental Protection Agency; 2017 [cited 2018. https://www.epa.gov/pesticide-science-and-assessingpesticide-risks/aquatic-life-benchmarks-and-ecological-risk.

[40] Sneck-Fahrer DA, East JW. Water-quality, sediment-quality, stream-habitat, and biological data for Mustang Bayou near Houston, Texas, 2004–05. U.S. Geological Survey, Interior USDot; 2007.

[41] Al-Badran AA, Fujiwara M, Gatlin III DM, Mora MA. Lethal and sub-lethal effects of the insecticide fipronil on juvenile brown shrimp Farfantepenaeus aztecus. Scientific Reports. 2018. 10.1038/s41598-018-29104-3 PMC6050305 30018298

[42] Lovell T. Nutrition and Feeding of Fish. 2nd ed New York, USA: Springer Science+Business Media, LLC; 1998.

[43] Kollman W, Randall S. Interim Report of the Pesticide Chemistry Database. Environmental Protection Agency. Department of Pesticide Regulation. 1020 N Street, Sacramento, California. 95814–5604. EH 95–04. 45 p; 1995.

[44] Lamers M, Anyusheva M, La N, Nguyen VV, Streck T. Pesticide Pollution in Surface- and Groundwater by Paddy Rice Cultivation: A Case Study from Northern Vietnam. CLEAN—Soil, Air, Water. 2011;39(4):356–61.

[45] Greenberg L, Rust MK, Klotz JH, Haver D, Kabashima JN, Bondarenko S, et al Impact of ant control technologies on insecticide runoff and efficacy. Pest Manag Sci. 2010;66(9):980–7. 10.1002/ps.1970 . 20730990

[46] Starner K, Goh KS. Detections of the neonicotinoid insecticide imidacloprid in surface waters of three agricultural regions of California, USA, 2010–2011. Bull Environ Contam Toxicol. 2012 3;88(3):316–21. 10.1007/s00128-011-0515-5 . 22228315

[47] USGS. Fipronil and degradation products in the rice-producing areas of the mermentau river basin, Louisiana, February–September 2000. Fact Sheet FS-010-03. US Geological Survey (USGS). 2003:6 p.

[48] Wirth EF, Pennington PL, Lawton JC, DeLorenzo ME, Bearden D, Shaddrix B, et al The effects of the contemporary-use insecticide (fipronil) in an estuarine mesocosm. Environ Pollut. 2004 10;131(3):365–71. 10.1016/j.envpol.2004.03.012 . 15261399

[49] Fossen M. Environmental Fate of Imidacloprid. Environmental Monitoring. Department of Pesticide Regulation. 1001 I Street. Sacramento, CA 95812–4015. 16 p; 2006.

[50] Gilliom RJ, Barbash JE, Crawford CG, Hamilton MC, Martin BT, Nakagaki N, et al. The Quality of Our Nation’s Waters—Pesticides in the Nation’s Streams and Ground Water, 1992–2001. U.S. Geological Survey Circular 1291. 2006:172 p.

[51] USEPA. Methods for measuring the acute toxicity of effluents and receiving waters to freshwater and marine organisms. United States Environmental Protection Agency. Office of Water. Washington DC. USA; 2002.

[52] Shan Z, Wang L, Cai D, Gong R, Zhu Z, Yu F. Impact of fipronil on crustacean aquatic organisms in a paddy field-fishpond ecosystem. Bull Environ Contam Toxicol. 2003;70(4):746–52. 10.1007/s00128-003-0046-9 . 12677386

[53] USEPA. Fipronil environmental fate and ecological effects assessment and characterization for section 18 registration of in-furrow applications to rutabaga and turnips. United States Environmental Protection Agency. Environmental Fate and Effects Division. 72 p (Washington DC. USA). 2007.

[54] JMP. JMP^®^ Pro. V. 13.1.0. SAS Institute Inc. Cary, North Carolina, U.S.A. 2016.

[55] Lassuy DR. Species profiles: Life histories and environmental requirements (Gulf of Mexico), brown shrimp. U.S. Fish and Wildlife Service. Coastal Engineering Research Center. 1983:15 P.

[56] Mensah PK, Palmer CG, Muller WJ. Lipid peroxidation in the freshwater shrimp Caridina nilotica as a biomarker of Roundup((R)) herbicide pollution of freshwater systems in South Africa. Water Sci Technol. 2012;65(9):1660–6. 10.2166/wst.2012.060 . 22508130

[57] Rozas LP, Minello TJ, Miles MS. Effect of deepwater horizon oil on growth rates of juvenile penaeid shrimps. Estuar Coast. 2014;37:1403–14.

[58] Stoughton SJ, Liber K, Culp J, Cessna A. Acute and chronic toxicity of imidacloprid to the aquatic invertebrates Chironomus tentans and Hyalella azteca under constant- and pulse-exposure conditions. Arch Environ Contam Toxicol. 2008 5;54(4):662–73. 10.1007/s00244-007-9073-6 . 18214581

[59] Azevedo-Pereira HMVS, Lemos MFL, Soares AMVM. Behaviour and Growth of Chironomus riparius Meigen (Diptera: Chironomidae) under Imidacloprid Pulse and Constant Exposure Scenarios. Water, Air, & Soil Pollution. 2011;219(1–4):215–24.

[60] Goff AD, Saranjampour P, Ryan LM, Hladik ML, Covi JA, Armbrust KL, et al The effects of fipronil and the photodegradation product fipronil desulfinyl on growth and gene expression in juvenile blue crabs, Callinectes sapidus, at different salinities. Aquat Toxicol. 2017;186:96–104. 10.1016/j.aquatox.2017.02.027 . 28282622

[61] Frontera JL, Vatnick I, Chaulet A, Rodriguez EM. Effects of glyphosate and polyoxyethylenamine on growth and energetic reserves in the freshwater crayfish Cherax quadricarinatus (Decapoda, Parastacidae). Arch Environ Contam Toxicol. 2011;61(4):590–8. 10.1007/s00244-011-9661-3 . 21424220

[62] Gauquelin F, Cuzon G, Gaxiola G, Rosas C, Arena L, Bureau DP, et al Effect of dietary protein level on growth and energy utilization by Litopenaeus stylirostris under laboratory conditions. Aquaculture. 2007;271(1–4):439–48.

[63] Ecobichon DJ. Toxic effects of pesticides In: Klaassen CD, editor. Casarett & Doull’s Toxicology: The Basic Science of Poisons. New York: McGraw-Hill; 1996 p. 782.

[64] Morrissey CA, Mineau P, Devries JH, Sanchez-Bayo F, Liess M, Cavallaro MC, et al Neonicotinoid contamination of global surface waters and associated risk to aquatic invertebrates: a review. Environ Int. 2015 1;74:291–303. 10.1016/j.envint.2014.10.024 . 25454246

[65] Hasenbein S, Lawler SP, Geist J, Connon RE. The use of growth and behavioral endpoints to assess the effects of pesticide mixtures upon aquatic organisms. Ecotoxicology. 2015;24(4):746–59. 10.1007/s10646-015-1420-1 . 25630500

[66] Minello TJ, Zimmerman RJ, Martinez EX. Mortality of Young Brown Shrimp Penaeus aztecus in Estuarine Nurseries. Transactions of the American Fisheries Society. 1989;118(6):693–708.

[67] Baker R, Fujiwara M, Minello TJ. Juvenile growth and mortality effects on white shrimp Litopenaeus setiferus population dynamics in the northern Gulf of Mexico. Fisheries Research. 2014;155:74–82.

[68] Lachaise F, Le Roux A, Hubert M, Lafont R. The molting gland of crustaceans: localization, activity, and endocrine control (Review). J Crustac Biol. 1993;13(2):198–234.

[69] OECD. Detaild review paper on aquatic Arthropods in life cycle and two-generation toxicity tests. Organisation for Economic Co-operation and Development. 2005 (50):135 p.

[70] Volz DC, Wirth EF, Fulton MH, Scott GI, Strozier E, Block DS, et al Effects of fipronil and chlorpyrifos on endocrine-related endpoints in female grass shrimp (Palaemonetes pugio). Bull Environ Contam Toxicol. 2003;71(3):497–503. 10.1007/s00128-003-8920-z 14567575

[71] Baines D, Wilton E, Pawluk A, de Gorter M, Chomistek N. Neonicotinoids act like endocrine disrupting chemicals in newly-emerged bees and winter bees. Sci Rep. 2017 9 8;7(1):10979 10.1038/s41598-017-10489-6 . 28887455 PMC5591280

[72] Key PB, Chung KW, Opatkiewicz AD, Wirth EF, Fulton MH. Toxicity of the insecticides fipronil and endosulfan to selected life stages of the grass shrimp (Palaemonetes pugio). Bull Environ Contam Toxicol. 2003;70(3):533–40. 10.1007/s00128-003-0019-z . 12592529

[73] Overmyer JP, Rouse DR, Avants JK, Garrison AW, DeLorenzo ME, Chung KW, et al Toxicity of fipronil and its enantiomers to marine and freshwater non-targets. J Environ Sci Health, Part B. 2007;42(5):471–80. 10.1080/03601230701391823 17562454

[74] Chaton PF, Ravanel P, Tissut M, Meyran JC. Toxicity and bioaccumulation of fipronil in the nontarget Arthropodan fauna associated with subalpine mosquito breeding sites. Ecotoxicol Environ Saf. 2002;52:8–12. 10.1006/eesa.2002.2166 12051802

[75] Leo JP, Minello TJ, Grant WE. Assessing Variability in Juvenile Brown Shrimp Growth Rates in Small Marsh Ponds: An Exercise in Model Evaluation and Improvement. Marine and Coastal Fisheries. 2018;10(3):347–56.

[76] Adamack AT, Stow CA, Mason DM, Rozas LP, Minello TJ. Predicting the effects of freshwater diversions on juvenile brown shrimp growth and production: a Bayesian-based approach. Marine Ecology Progress Series. 2012;444:155–73.

[77] Zein-Eldin ZP, Aldrich DV. Growth and Survival of Postlarval Penaeus aztecus under Controlled Conditions of Temperature and Salinity. Biological Bulletin. 1965;129(1):199–216.

[78] Russo R, Becker JM, Liess M. Sequential exposure to low levels of pesticides and temperature stress increase toxicological sensitivity of crustaceans. The Science of the total environment. 2018 1 1;610–611:563–9. 10.1016/j.scitotenv.2017.08.073 . 28822923

[79] Willming MM, Qin G, Maul JD. Effects of environmentally realistic daily temperature variation on pesticide toxicity to aquatic invertebrates. Environ Toxicol Chem. 2013 12;32(12):2738–45. 10.1002/etc.2354 . 23955707

[80] Tu HT, Silvestre F, Phuong NT, Kestemont P. Effects of pesticides and antibiotics on penaeid shrimp with special emphases on behavioral and biomarker responses. Environ Toxicol Chem. 2010 4;29(4):929–38. 10.1002/etc.99 . 20821523

[81] Raley-Susman KM. Like a Canary in the coal mine: behavioral change as an early warning sign of neurotoxicological damage. In: Soloneski S, editor. Pesticides—Toxic Aspects inTech; 2014. p. 29 p.

[82] Stratman KN, Wilson PC, Overholt WA, Cuda JP, Netherland MD. Toxicity of fipronil to the midge, Cricotopus lebetis Sublette. J Toxicol Environ Health, Part A. 2013;76(12):716–22. 10.1080/15287394.2013.802266 . 23980838

[83] Roque A, Abad S, Betancourt-Lozano M, de la Parra LM, Baird D, Guerra-Flores AL, et al Evaluation of the susceptibility of the cultured shrimp Litopenaeus vannamei to vibriosis when orally exposed to the insecticide methyl parathion. Chemosphere. 2005;60(1):126–34. 10.1016/j.chemosphere.2005.01.008 . 15910911

[84] Martinez A, Romero Y, Castillo T, Mascaro M, Lopez-Rull I, Simoes N, et al The effect of copper on the color of shrimps: redder is not always healthier. PloS one. 2014;9(9):e107673 10.1371/journal.pone.0107673 . 25229639 PMC4167854

[85] O’Halloran MJ. Color control in shrimp. In: Goldman CA, editor. Tested Studies for Laboratory Teaching. 111990. p. 15–26.

[86] Fernlund P, Josefsson L. Crustacean color-change hormone: amino acid sequence and chemical synthesis. Science. 1972;177(4044):173–5. 10.1126/science.177.4044.173 5041363

[87] Fingerman M, Jackson NC, Nagabhushanam R. Hormonally-regulated functions in crustaceans as biomarkers of environmental pollution (Review). Comp Biochem Physiol Part C. 1998;120:343–50. 10.1016/s0742-8413(98)10072-5 9827049

