# Supplementary material for: Effects of insecticides, fipronil and imidacloprid, on the growth, survival, and behavior of brown shrimp Farfantepenaeus aztecus
Source: PLoS One. 2019 Oct 10;14(10):e0223641. doi: 10.1371/journal.pone.0223641 (PMC6786580; doi:10.1371/journal.pone.0223641)
Supplement: S1 Fig — (A) System of 18 glass aquariums covered with aluminum foil sheets and glass lids; (B) aquarium was divided into six cells of same size; (C) dividers made of polypropylene plates and fiberglass screen to distribute the dissolved oxygen among the cells; (D) total length of shrimp measured every week during the experiments. (DOCX) [file pone.0223641.s001.docx]

Effects of insecticides, fipronil and imidacloprid, on the growth, survival, and behavior of brown shrimp *Farfantepenaeus aztecus*

**Ali Abdulameer Al-Badran^1*^, Masami Fujiwara^1^, Miguel A. Mora^1^**

1. Department of Wildlife and Fisheries Sciences, Texas A&M University, College Station, Texas, United States of America

* Corresponding author

E-mail: [aliabdulameer33@gmail.com](mailto:*aliabdulameer33@gmail.com) (AA)


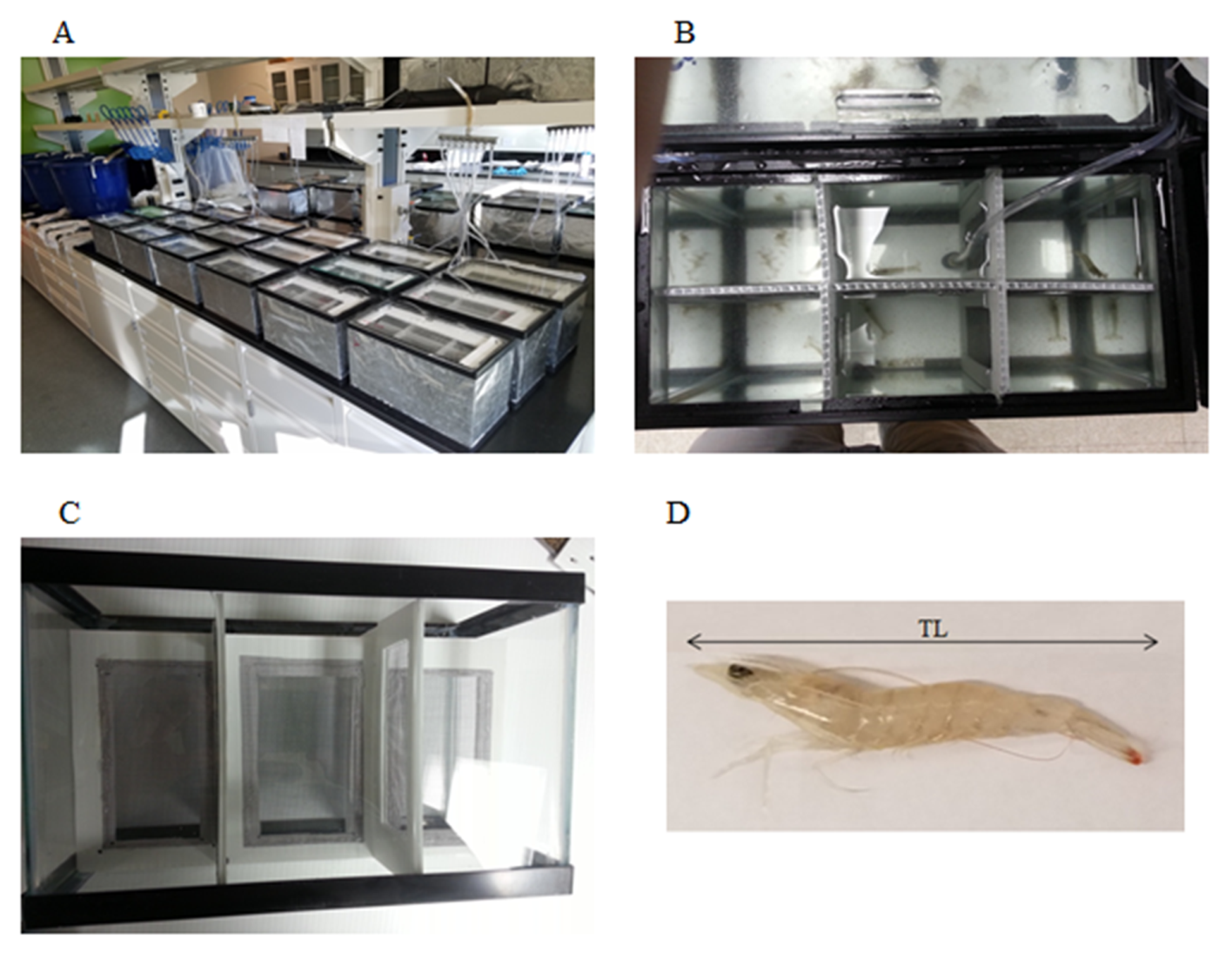


**S1 Fig. Experimental systems and the aquariums used in fipronil and imidacloprid experiments:**

(**A**) System of 18 glass aquariums covered with aluminum foil sheets and glass lids; (**B**) aquarium was divided into six cells of same size; (**C**) dividers made of polypropylene plates and fiberglass screen to distribute the dissolved oxygen among the cells; (**D**) total length of shrimp measured every week during the experiments.
