# Supplementary material for: Effects of insecticides, fipronil and imidacloprid, on the growth, survival, and behavior of brown shrimp Farfantepenaeus aztecus
Source: PLoS One. 2019 Oct 10;14(10):e0223641. doi: 10.1371/journal.pone.0223641 (PMC6786580; doi:10.1371/journal.pone.0223641)
Supplement: S2 Fig — (DOCX) [file pone.0223641.s002.docx]

Effects of insecticides, fipronil and imidacloprid, on the growth, survival, and behavior of brown shrimp *Farfantepenaeus aztecus*

**Ali Abdulameer Al-Badran^1*^, Masami Fujiwara^1^, Miguel A. Mora^1^**

1. Department of Wildlife and Fisheries Sciences, Texas A&M University, College Station, Texas, United States of America

* Corresponding author

E-mail: [aliabdulameer33@gmail.com](mailto:*aliabdulameer33@gmail.com) (AA)


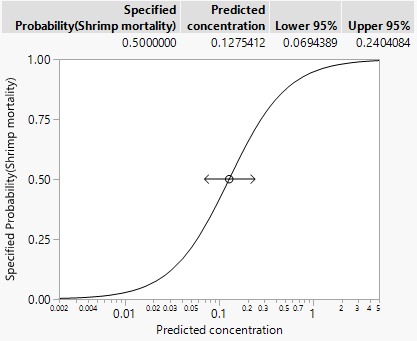


**S2 Fig. Lethal concentration of fipronil to reach 50% mortality of shrimp within 96 hours (96-h LC_50_) of the juvenile brown shrimp**.
