# Supplementary material for: Effects of insecticides, fipronil and imidacloprid, on the growth, survival, and behavior of brown shrimp Farfantepenaeus aztecus
Source: PLoS One. 2019 Oct 10;14(10):e0223641. doi: 10.1371/journal.pone.0223641 (PMC6786580; doi:10.1371/journal.pone.0223641)
Supplement: S1 Table — For steps 1 and 2, magnetic stirrer was used to homogenize the mixture. (DOCX) [file pone.0223641.s003.docx]

Effects of insecticides, fipronil and imidacloprid, on the growth, survival, and behavior of brown shrimp *Farfantepenaeus aztecus*

**Ali Abdulameer Al-Badran^1*^, Masami Fujiwara^1^, Miguel A. Mora^1^**

1. Department of Wildlife and Fisheries Sciences, Texas A&M University, College Station, Texas, United States of America

* Corresponding author

E-mail: [aliabdulameer33@gmail.com](mailto:*aliabdulameer33@gmail.com) (AA)

**S1 Table. Dilution procedures for all nominal fipronil concentrations used in the experiment**.

For steps 1 and 2, magnetic stirrer was used to homogenize the mixture.

| **Fipronil concentration**  **µg/L** | **Dilution steps** | | |
| --- | --- | --- | --- |
|  | **Step 1** | **Step 2** | **Step 3** |
|  | **100 mg/L Fipronil**  **suspension** | **0.1 mg/L Fipronil**  **solution** |  |
| 0.005 | Mix 0.1 g of fipronil powder in 1000 ml of brackish water | Mix 1.0 ml of 100 mg/L fipronil suspension in 999 ml of brackish water | Mix 10 ml of 0.1 mg/L fipronil solution in 990 ml of water to make 1.0 µg/L fipronil solution, then mix 105 ml of 1.0 µg/L fipronil in (21,000 ml – 105 ml) of water |
| 0.01 |  |  | Mix 10 ml of 0.1 mg/L fipronil solution in 990 ml of water to make 1.0 µg/L fipronil solution, then mix 210 ml of 1.0 µg/Lfipronil in (21,000 ml – 210 ml) of water |
| 0.1 |  |  | Mix 21 ml of 0.1 mg/L fipronil solution in (21,000 ml – 21 ml) of water |
| 1.0 |  |  | Mix 210 ml of 0.1 mg/L fipronil solution in (21,000 ml – 210 ml) of water |
| 3.0 |  |  | Mix 630 ml of 0.1 mg/L fipronil solution in (21,000 ml – 630 ml) of water |
