# Supplementary material for: Effects of insecticides, fipronil and imidacloprid, on the growth, survival, and behavior of brown shrimp Farfantepenaeus aztecus
Source: PLoS One. 2019 Oct 10;14(10):e0223641. doi: 10.1371/journal.pone.0223641 (PMC6786580; doi:10.1371/journal.pone.0223641)
Supplement: S2 Table — For steps 1 and 2, magnetic stirrer was used to homogenize the mixture. (DOCX) [file pone.0223641.s004.docx]

Effects of insecticides, fipronil and imidacloprid, on the growth, survival, and behavior of brown shrimp *Farfantepenaeus aztecus*

**Ali Abdulameer Al-Badran^1*^, Masami Fujiwara^1^, Miguel A. Mora^1^**

1. Department of Wildlife and Fisheries Sciences, Texas A&M University, College Station, Texas, United States of America

* Corresponding author

E-mail: [aliabdulameer33@gmail.com](mailto:*aliabdulameer33@gmail.com) (AA)

**S2 Table**. **Dilution procedures for all nominal imidacloprid concentrations used in the experiment**.

For steps 1 and 2, magnetic stirrer was used to homogenize the mixture.

| **Imidacloprid concentration**  **µg/L** | **Dilution steps** | | |
| --- | --- | --- | --- |
|  | **Step 1** | **Step 2** | **Step 3** |
|  | **10 mg/L Imidacloprid**  **solution** | **1000µg/L Imidacloprid**  **solution** |  |
| 0.5 | Mix 0.01 g of Imidacloprid powder in 1000 ml of brackish water | Mix 100 ml of 10 mg/L Imidacloprid  solution in 900 ml of brackish water | Mix 10.5 ml of 1000 µg/L Imidacloprid  solution in (21,000 ml – 10.5 ml) of water |
| 1.0 |  |  | Mix 21 ml of 1000 µg/L Imidacloprid  solution in (21,000 ml – 21 ml) of water |
| 15.0 |  |  | Mix 315 ml of 1000 µg/L Imidacloprid  solution in (21,000 ml – 315 ml) of water |
| 34.5 |  |  | Mix 724.5 ml of 1000 µg/L Imidacloprid  solution in (21,000 ml – 724.5 ml) of water |
| 320.0 |  |  | Mix 67.2 ml of 10mg/L Imidacloprid  solution in (21,000 ml – 67.2 ml) of water |
