# Supplementary material for: Effects of insecticides, fipronil and imidacloprid, on the growth, survival, and behavior of brown shrimp Farfantepenaeus aztecus
Source: PLoS One. 2019 Oct 10;14(10):e0223641. doi: 10.1371/journal.pone.0223641 (PMC6786580; doi:10.1371/journal.pone.0223641)
Supplement: S4 Table — n = number of shrimp in each treatment at the measurement time. Values were calculated based on the wet weight per individual shrimp. Means in columns not sharing the same letter are significantly different (ANOVA, P < 0.05). (DOCX) [file pone.0223641.s006.docx]

Effects of insecticides, fipronil and imidacloprid, on the growth, survival, and behavior of brown shrimp *Farfantepenaeus aztecus*

**Ali Abdulameer Al-Badran^1*^, Masami Fujiwara^1^, Miguel A. Mora^1^**

1. Department of Wildlife and Fisheries Sciences, Texas A&M University, College Station, Texas, United States of America

* Corresponding author

E-mail: [aliabdulameer33@gmail.com](mailto:*aliabdulameer33@gmail.com) (AA)

**S4 Table.** **Initial weight (g), final weight (g), and percent weight gain (mean ± standard deviation) of juvenile shrimp under different concentrations of imidacloprid**.

n = number of shrimp in each treatment at the measurement time. Values were calculated based on the wet weight per individual shrimp. Means in columns not sharing the same letter are significantly different (ANOVA, P ˂ 0.05).

| **Imidacloprid concentrations (µg/L)** | **Initial weight (g)** | **n** | **Final weight (g)** | **n** | **% Weight gain** |
| --- | --- | --- | --- | --- | --- |
| Control | 0.81 ± 0.03  **a** | 15 | 1.95 ± 0.12  **a** | 15 | 140.3 ± 16.15  **a** |
| 0.5 | 0.84 ± 0.11  **a** | 15 | 1.87 ± 0.13  **ab** | 14 | 126.89 ± 46.88  **a** |
| 1.0 | 0.81 ± 0.03  **a** | 15 | 1.65 ± 0.18  **b** | 13 | 103.39 ± 27.92  **ab** |
| 15.0 | 0.80 ± 0.06  **a** | 15 | 1.31 ± 0.12  **c** | 10 | 64.40 ± 17.14  **bc** |
| 34.5 | 0.84 ± 0.06  **a** | 15 | 1.21 ± 0.05  **cd** | 6 | 44.01 ± 12.09  **c** |
| 320.0 | 0.80 ± 0.09  **a** | 15 | 1.04 ± 0.13  **d** | 5 | 29.48 ± 16.43  **c** |
