# Supplementary material for: Effects of insecticides, fipronil and imidacloprid, on the growth, survival, and behavior of brown shrimp Farfantepenaeus aztecus
Source: PLoS One. 2019 Oct 10;14(10):e0223641. doi: 10.1371/journal.pone.0223641 (PMC6786580; doi:10.1371/journal.pone.0223641)
Supplement: S5 Table — n = number of shrimp in each treatment. Means in columns not sharing the same letter are significantly different (ANOVA, P < 0.05). (DOCX) [file pone.0223641.s007.docx]

Effects of insecticides, fipronil and imidacloprid, on the growth, survival, and behavior of brown shrimp *Farfantepenaeus aztecus*

**Ali Abdulameer Al-Badran^1*^, Masami Fujiwara^1^, Miguel A. Mora^1^**

1. Department of Wildlife and Fisheries Sciences, Texas A&M University, College Station, Texas, United States of America

* Corresponding author

E-mail: [aliabdulameer33@gmail.com](mailto:*aliabdulameer33@gmail.com) (AA)

**S5 Table. Length (cm) of juvenile shrimp (mean ± standard deviation) exposed to fipronil over five weeks**.

n = number of shrimp in each treatment. Means in columns not sharing the same letter are significantly different (ANOVA, P ˂ 0.05).

| **Fipronil concentrations (µg/L)** | **Initial length (cm)** | **Length week 1**  **(cm)** | **Length week 2**  **(cm)** | **Lengthweek 3**  **(cm)** | **Length week 4**  **(cm)** | **Lengthweek 5**  **(cm)** |
| --- | --- | --- | --- | --- | --- | --- |
| **Control** | 4.46 ± 0.09 (n = 18)  a | 4.84 ± 0.09 (n = 18)  a | 5.25 ± 0.05 (n = 18)  a | 5.62 ± 0.05 (n = 18)  a | 5.98 ± 0.12 (n = 18)  a | 6.32 ± 0.10 (n = 18)  a |
| **0.005** | 4.44 ± 0.01 (n = 18)  a | 4.79 ± 0.02 (n = 18)  ab | 5.07 ± 0.07 (n = 18)  a | 5.50 ± 0.04 (n = 18)  ab | 5.91 ± 0.05 (n = 18)  ab | 6.32 ± 0.03 (n = 18)  a |
| **0.01** | 4.38 ± 0.11 (n = 18)  a | 4.64 ± 0.15 (n = 14)  bc | 5.02 ± 0.18 (n = 14)  a | 5.27 ± 0.08 (n = 13)  bc | 5.69 ± 0.13 (n = 13)  bc | 5.99 ± 0.16 (n = 13)  ab |
| **0.1** | 4.43 ± 0.03 (n = 18)  a | 4.58 ± 0.10 (n = 8)  c | 4.63 ± 0.24 (n = 6)  b | 5.05 ± 0.25 (n = 6)  c | 5.49 ± 0.18 (n = 6)  c | 5.87 ± 0.33 (n = 6)  b |
| **1.0** | 4.37 ± 0.15 (n = 18)  a | / | / | / | / | / |
| **3.0** | 4.39 ± 0.07 (n = 18)  a | / | / | / | / | / |
