# Supplementary material for: Effects of insecticides, fipronil and imidacloprid, on the growth, survival, and behavior of brown shrimp Farfantepenaeus aztecus
Source: PLoS One. 2019 Oct 10;14(10):e0223641. doi: 10.1371/journal.pone.0223641 (PMC6786580; doi:10.1371/journal.pone.0223641)
Supplement: S6 Table — n = number of shrimp in each treatment. Means in columns not sharing the same letter are significantly different (ANOVA, P < 0.05). (DOCX) [file pone.0223641.s008.docx]

Effects of insecticides, fipronil and imidacloprid, on the growth, survival, and behavior of brown shrimp *Farfantepenaeus aztecus*

**Ali Abdulameer Al-Badran^1*^, Masami Fujiwara^1^, Miguel A. Mora^1^**

1. Department of Wildlife and Fisheries Sciences, Texas A&M University, College Station, Texas, United States of America

* Corresponding author

E-mail: [aliabdulameer33@gmail.com](mailto:*aliabdulameer33@gmail.com) (AA)

**S6 Table. Length (cm) of juvenile shrimp (mean ± standard deviation) exposed to imidacloprid over five weeks**.

n = number of shrimp in each treatment. Means in columns not sharing the same letter are significantly different (ANOVA, P ˂ 0.05).

| **Imidacloprid concentrations (µg/L)** | **Initial length (cm)** | **Lengthweek 1**  **(cm)** | **Lengthweek 2**  **(cm)** | **Lengthweek 3**  **(cm)** | **Lengthweek 4**  **(cm)** | **Lengthweek 5**  **(cm)** |
| --- | --- | --- | --- | --- | --- | --- |
| **Control** | 5.28 ± 0.1 (n = 15)  a | 5.83 ± 0.09 (n = 15)  a | 6.22 ± 0.09 (n = 15)  a | 6.57 ± 0.09 (n = 15)  a | 6.92 ± 0.17 (n = 15)  a | 7.07 ± 0.12(n = 15)  a |
| **0.5** | 5.32 ± 0.09(n = 15)  a | 5.8 ± 0.21(n = 15)  a | 6.17 ± 0.2(n = 14)  ab | 6.55 ± 0.22(n = 14)  a | 6.77 ± 0.2(n = 14)  ab | 6.96 ± 0.18(n = 14)  a |
| **1.0** | 5.33 ± 0.15(n = 15)  a | 5.67 ± 0.13(n = 14)  a | 5.92 ± 0.26(n = 14)  abc | 6.28 ± 0.27(n = 14)  a | 6.48 ± 0.31(n = 14)  bc | 6.72 ± 0.24(n = 13)  a |
| **15.0** | 5.28 ± 0.21(n = 15)  a | 5.59 ± 0.15(n = 15)  a | 5.79 ± 0.18(n = 15)  bc | 5.86 ± 0.24(n = 15)  b | 6.12 ± 0.2(n = 11)  cd | 6.25 ± 0.24(n = 10)  b |
| **34.5** | 5.38 ± 0.11(n = 15)  a | 5.52 ± 0.09(n = 15)  a | 5.58 ± 0.13(n = 15)  c | 5.7 ± 0.08(n = 11)  b | 5.79 ± 0.11(n = 9)  d | 5.98 ± 0.05(n = 6)  b |
| **320.0** | 5.29 ± 0.2(n = 15)  a | 5.63 ± 0.29(n = 15)  a | 5.66 ± 0.35(n = 15)  c | 5.82 ± 0.32(n = 12)  b | 5.97 ± 0.31(n = 9)  d | 5.91 ± 0.23(n = 5)  b |
