# Supplementary material for: Effects of insecticides, fipronil and imidacloprid, on the growth, survival, and behavior of brown shrimp Farfantepenaeus aztecus
Source: PLoS One. 2019 Oct 10;14(10):e0223641. doi: 10.1371/journal.pone.0223641 (PMC6786580; doi:10.1371/journal.pone.0223641)
Supplement: S7 Table — Values are Mean ± standard deviation for each parameter of all fipronil concentrations. Treatment 1.0 μg/L has no standard deviation because the number of aquariums was reduced to 1 due to deaths of shrimp during first days of the experiment. Treatment 3.0 μg/L has no water quality parameters because all shrimp died during the first day of the exposure. (DOCX) [file pone.0223641.s009.docx]

Effects of insecticides, fipronil and imidacloprid, on the growth, survival, and behavior of brown shrimp *Farfantepenaeus aztecus*

**Ali Abdulameer Al-Badran^1*^, Masami Fujiwara^1^, Miguel A. Mora^1^**

1. Department of Wildlife and Fisheries Sciences, Texas A&M University, College Station, Texas, United States of America

* Corresponding author

E-mail: [aliabdulameer33@gmail.com](mailto:*aliabdulameer33@gmail.com) (AA)

**S7 Table**. **Water quality parameters of shrimp aquariums during 34 days of fipronil experiment**.

Values are Mean ± standard deviation for each parameter of all fipronil concentrations. Treatment 1.0 µg/L has no standard deviation because the number of aquariums was reduced to 1 due to deaths of shrimp during first days of the experiment. Treatment 3.0 µg/L has no water quality parameters because all shrimp died during the first day of the exposure.

| **Fipronil concentrations**  **(µg/L)** | **Water quality parameters** | | | |
| --- | --- | --- | --- | --- |
|  | **Temp. °C** | **DO mg/L** | **Salinity ‰** | **pH** |
| **Control** | 24.24 ± 0.18 | 5.32 ± 0.59 | 15.16 ± 0.37 | 7.90 ± 0.17 |
| **0.005** | 24.08 ± 0.16 | 5.67 ± 0.27 | 14.95 ± 0.78 | 8.02 ± 0.13 |
| **0.01** | 24.07 ± 0.19 | 5.56 ± 0.49 | 15.12 ± 0.42 | 8.02 ± 0.12 |
| **0.1** | 24.14 ± 0.15 | 6.08 ± 0.25 | 15.14 ± 0.39 | 8.07 ± 0.12 |
| **1.0** | 24.10 | 6.5 | 14.77 | 8.07 |
| **3.0** | / | / | / | / |
