# Supplementary material for: Effects of insecticides, fipronil and imidacloprid, on the growth, survival, and behavior of brown shrimp Farfantepenaeus aztecus
Source: PLoS One. 2019 Oct 10;14(10):e0223641. doi: 10.1371/journal.pone.0223641 (PMC6786580; doi:10.1371/journal.pone.0223641)
Supplement: S8 Table — Values are Mean ± standard deviation for each parameter of all imidacloprid concentrations. (DOCX) [file pone.0223641.s010.docx]

Effects of insecticides, fipronil and imidacloprid, on the growth, survival, and behavior of brown shrimp *Farfantepenaeus aztecus*

**Ali Abdulameer Al-Badran^1*^, Masami Fujiwara^1^, Miguel A. Mora^1^**

1. Department of Wildlife and Fisheries Sciences, Texas A&M University, College Station, Texas, United States of America

* Corresponding author

E-mail: [aliabdulameer33@gmail.com](mailto:*aliabdulameer33@gmail.com) (AA)

**S8 Table**. **Water quality parameters of shrimp aquariums during 36 days of imidacloprid experiment**.

Values are Mean ± standard deviation for each parameter of all imidacloprid concentrations.

| **Imidacloprid concentrations**  **(µg/L)** | **Water quality parameters** | | | |
| --- | --- | --- | --- | --- |
|  | **Temp. °C** | **DO mg/L** | **Salinity ‰** | **pH** |
| **Control** | 24.34 ± 0.19 ^a^ | 5.86 ± 0.52 | 15.51 ± 0.34 | 8.08 ± 0.09 |
| **0.5** | 24.4 ± 0.15 | 5.9 ± 0.29 | 15.36 ± 0.12 | 8.10 ± 0.05 |
| **1.0** | 24.32 ± 0.16 | 5.8 ± 0.26 | 15.39 ± 0.15 | 8.12 ± 0.04 |
| **15.0** | 24.34 ± 0.11 | 5.94 ± 0.75 | 15.60 ± 0.12 | 8.20 ± 0.04 |
| **34.5** | 24.34 ± 0.16 | 5.8 ± 0.26 | 15.53 ± 0.14 | 8.19 ± 0.06 |
| **320.0** | 24.3 ± 0.12 | 5.96 ± 0.48 | 15.49 ± 0.11 | 8.22 ± 0.07 |
